# Systemic-to-pulmonary artery shunt treated with transcatheter arterial embolization and subsequent lung segmentectomy

**DOI:** 10.1186/s43044-023-00431-9

**Published:** 2023-12-21

**Authors:** Hirotsugu Notsuda, Fumiko Tomiyama, Ken Onodera, Tatsuaki Watanabe, Yui Watanabe, Hisashi Oishi, Hiromichi Niikawa, Chihiro Inoue, Hideki Ota, Masafumi Noda, Yoshinori Okada

**Affiliations:** 1 Department of Thoracic Surgery, Institute of Development, Aging and Cancer, Tohoku University Hospital, Tohoku University, 4-1, Seiryomachi, Aoba-ku, Sendai, Miyagi 980-8575 Japan; 2 Department of Anatomic Pathology, Tohoku University Graduate School of Medicine, Sendai, Japan; 3 Department of Diagnostic Radiology, Tohoku University Hospital, Sendai, Japan

**Keywords:** Systemic-to-pulmonary artery shunt (SPAS), Transcatheter arterial embolization (TAE), Surgical treatment, Segmentectomy, Case report

## Abstract

**Background:**

Systemic-to-pulmonary artery shunt (SPAS) is a rare condition that can occur as a result of congenital heart disease or chronic pulmonary inflammation, occasionally leading to life-threatening hemoptysis. Computed tomography (CT) imaging is crucial in the diagnosis of SPAS, and the optimal management approach for SPAS remains uncertain. This case report presents a novel approach to the treatment of SPAS, consisting of transcatheter arterial embolization of the systemic artery followed by lung segmentectomy.

**Case presentation:**

A 42-year-old man with abnormal chest findings was referred to us and a diagnosis of SPAS was established based on the CT findings showing a blood flow regurgitation from the dilated left 4th intercostal artery to the Lt. A6. The patient was asymptomatic but we decided to treat him to prevent a risk of future hemoptysis. Transcatheter arterial embolization (TAE) of systemic arteries followed by S6 segmentectomy was successfully performed with minimal blood loss and complete removal of the dilated intra-pulmonary blood vessels. Histological analysis confirmed the diagnosis of SPAS.

**Conclusion:**

We reported a case of SPAS, who was successfully treated with the combination of TAE and subsequent segmentectomy. The blood loss during surgery was minimal and this strategy appeared to minimize future recanalization and hemoptysis. Further studies and long-term follow-up of SPAS patients are required to establish standardized management guidelines for this rare condition.

## Background

Systemic-to-pulmonary artery sunt (SPAS) is a rare condition that can occur as a result of congenital heart disease or chronic pulmonary inflammation, leading to a life-threatening hemoptysis [1–3]. Computed tomography (CT) scan findings such as pleural thickness and enhancing vascular structures are suggestive of systemic arterial supply [4]. In patients with SPAS, there is regurgitation from the systemic arteries to the pulmonary arteries, as observed on contrast-enhanced CT scans. Transcatheter arterial embolization (TAE) has emerged as an important treatment modality for patients with SPAS. However, the optimal management for patients with SPAS remains uncertain, as TAE alone may provide temporary relief and subsequent recanalization or development of collateral neovascularization sometimes occur [4]. In this unique case report, we present a novel approach to treat SPAS consisting of TAE of the systemic artery followed by a lung segmentectomy.

## Case presentation

**Fig. 1:**
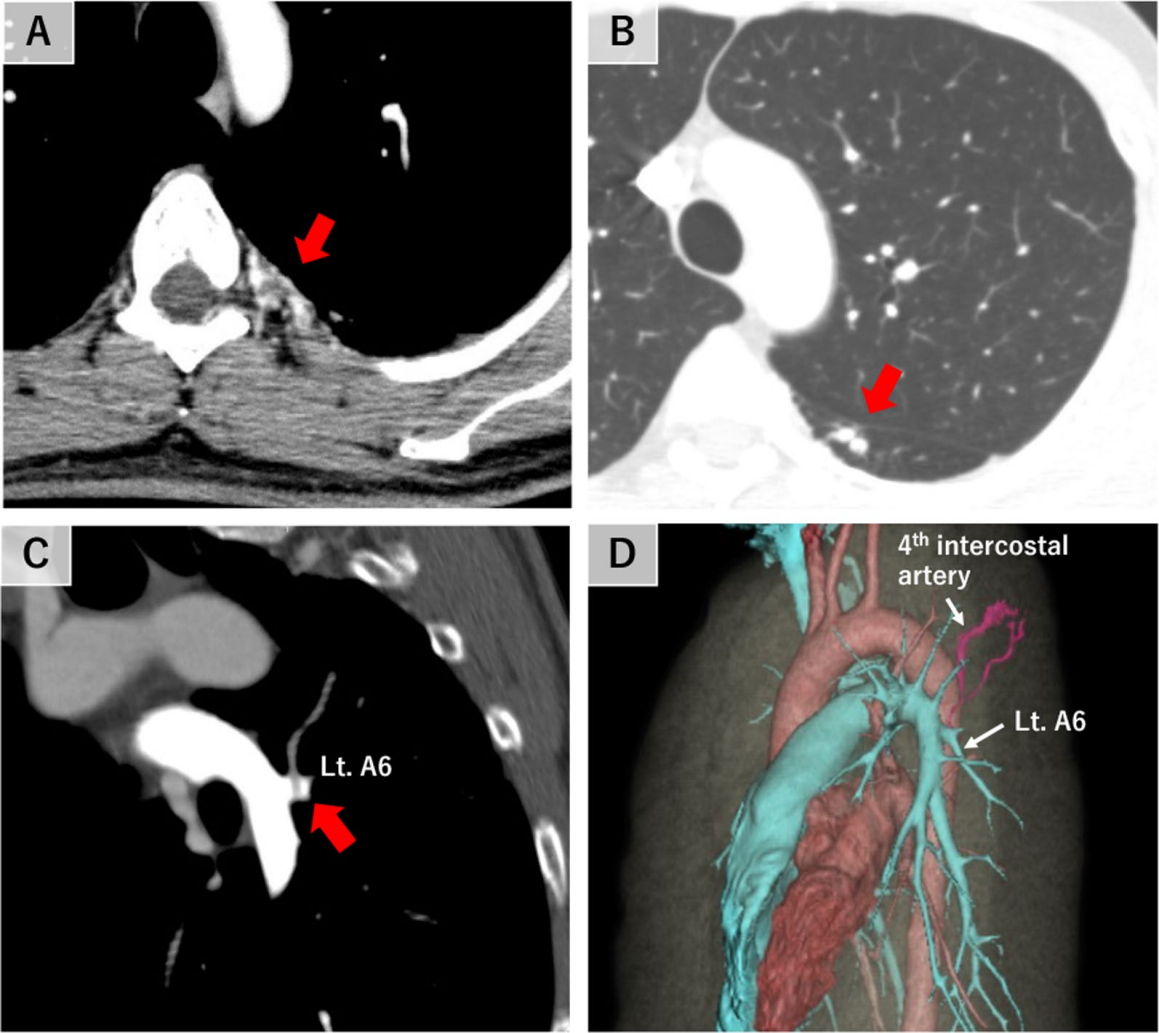
Computed tomographic images of the retrograde systemic to pulmonary artery shunt. **A** Numerous neovascular vessels in the left chest wall (red arrow). **B** Anomalous arteries in the left lower lobe superior segment of lung (Lt. S6, red arrow). **C** Filling defect in the left apical segmental pulmonary artery of the left lower lobe (Lt. A6, red arrow). **D** In the three-dimension (3D) image, the 4th intercostal artery, a systemic artery, flowed into the left apical segmental pulmonary artery of the left lower lobe (Lt. A6), which was diagnosed as the systemic-to-pulmonary artery shunt (SPAS)

**Fig. 2:**
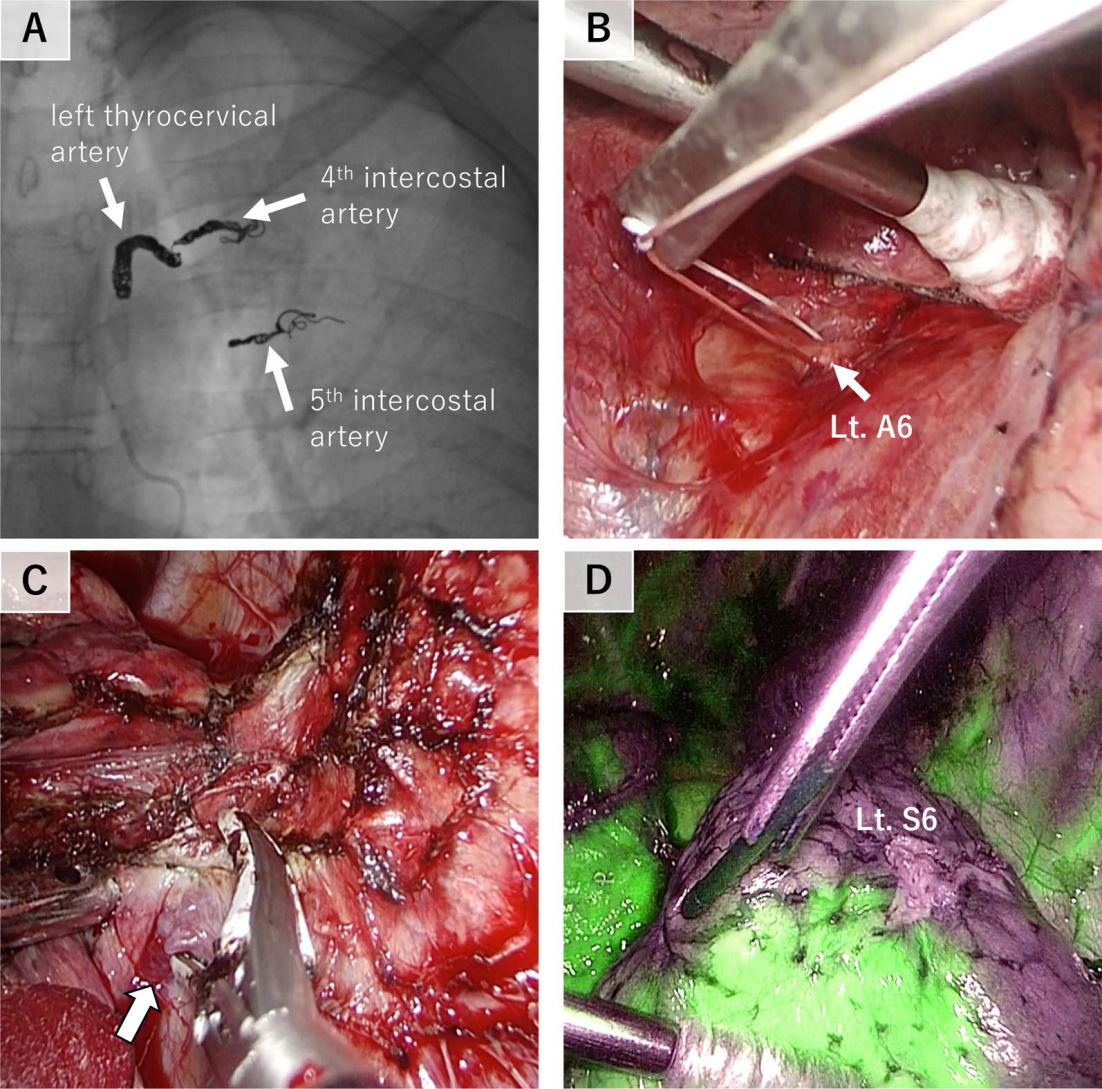
Perioperative embolization of systemic arteries and intraoperative photos **A** Preoperative transcatheter arterial embolization (TAE) of the intercostal artery and left thyrocervical artery. **B** Ligation of the left apical segmental pulmonary artery of the left lower lobe (Lt. A6). **C** Adhesion detachment and coagulation of neovascular vessels (white arrow). **D** Staining of lung areas by the indocyanine green (ICG), the left lower lobe superior segment of lung (Lt. S6) is observed as a poorly stained area

**Fig. 3:**
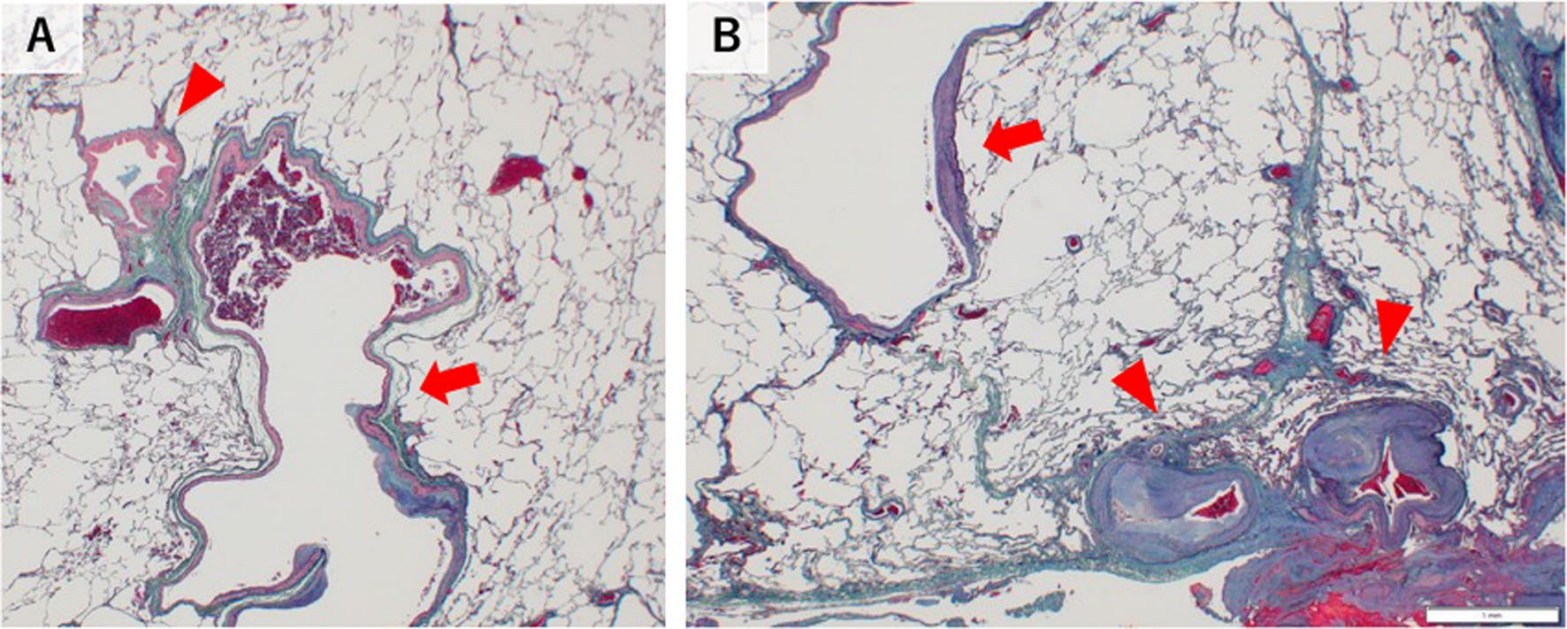
Pathological findings of the resected left S6 **A** Dilated pulmonary artery (red arrow) accompanying a normal bronchiole (red arrowhead). **B** Large muscular arteries (red arrowhead) and dilated pulmonary arteries (red arrow) are seen in the subpleural area

A 42-year-old man was referred to our hospital due to an abnormal shadow on a chest X-ray, which was found during a physical examination. CT scan revealed numerous vascular structures in the left chest wall (Fig. 1A), anomalous arteries in the left lower lobe superior segment of lung (Lt. S6) (Fig. 1B), and a filling defect in the left apical segmental pulmonary artery of the left lower lobe (Lt. A6), which indicated a blood flow regurgitation from the dilated left 4th intercostal artery to the Lt. A6 (Fig. 1C, D). No other abnormalities were found by CT scan, laboratory tests and pulmonary function test, and a diagnosis of SPAS was established. The patients was asymptomatic, but based on the potential risk of future hemoptysis, we decided to treat him with TAE followed by S6 segmentectomy after a careful informed consent. On preoperative TAE, the 4th and 5th intercostal arteries and the left thyrocervical artery were identified as systemic arteries and all were embolized with microcoils (Fig. 2A). After the TAE, S6 segmentectomy was performed on the same day. First, interlobar fissure was dissected and A6 was ligated and transected to reduce blood flow of the pulmonary artery, followed by the dissection of the adhesions between S6 and parietal pleura, containing abundant blood vessels (Fig. 2C). Finally, Lt. S6 segmentectomy was performed using indocyanine green (ICG) (Fig. 2D). Intraoperative blood loss was 30 g. The patient had an uneventful postoperative course, and he was discharged on the 6th day after surgery. Histological examination of the resected left S6 showed a pleural thickening and large muscular arteries and adjacent dilated pulmonary arteries in the subpleural area, suggesting communications between them (Fig. 3A, B).

## Discussion

SPASs, which develop in the absence of chronic pulmonary inflammatory disease or complex congenital cardiac anomalies, are considered to occur as a result of local inflammation such as trauma or infection [8, 9]. The bronchial arteries are the most common arteries involved in SPAS, but other non-bronchial arteries such as internal thoracic, intercostal, and inferior phrenic arteries can also contribute as shunting blood vessels into the pulmonary arteries [5].

CT imaging is crucial in the diagnosis of SPAS. It helps identify characteristic findings such as pleural thickness, neovascular vessels in the chest wall, aneurysms within the lung parenchyma, and filling defects in PA in the pulmonary-arterial phase [4, 10, 11].

In our case, CT scans revealed numerous neovascular vessels in the chest wall, a confluence of the left 4th intercostal artery and A6 and a filling defect in the left A6, which lead to the diagnosis of SPAS. A contrast radiographic study on TAE further identified intercostal arteries and left thyrocervical artery as the systemic arteries involved. The patient did not have congenital cardiovascular malformations or chronic respiratory disease, and a careful review of the patient's medical history failed to identify episode of trauma or local inflammation which can be underlying cause of SPAS.

Treatment strategies for SPAS are not well established, and there is no consensus or guidelines regarding the optimal management approach. Non-surgical options include embolization of the systemic arteries using TAE techniques [7, 12]. However, there is a risk of vessel recanalization and the development of collateral vessels [6]. Surgical treatments such as ligation of neovascular vessels at the chest wall, segmentectomy, lobectomy, or pneumonectomy have also be reported [9, 12, 13]. On the other hand, some reports insist that these lesions can be managed conservatively without intervention during long-term follow-up [1].

**Fig. 4:**
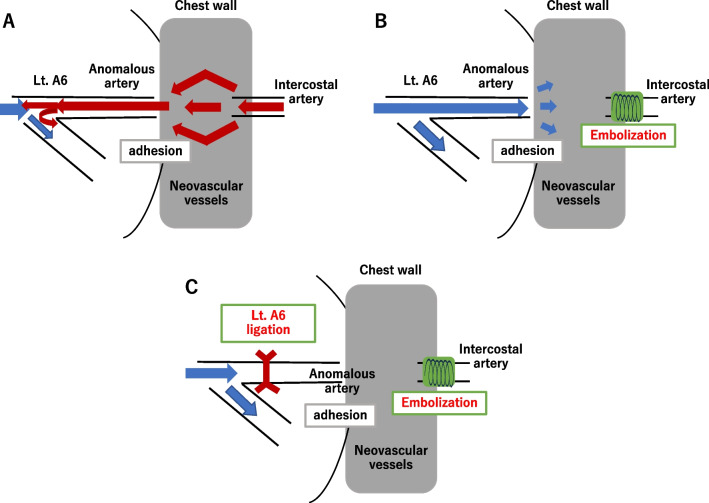
Schematic representations of surgical treatment **A** Before treatments, blood flow from systemic arteries to the left apical segmental pulmonary artery of the left lower lobe (Lt. A6) is active. **B** After embolization of two intercostal arteries and left thyrocervical artery, blood flow from the pulmonary artery was maintained. **C** After ligation of the left apical segmental pulmonary artery of the left lower lobe (Lt. A6), the blood flow from both sides were completely blocked

We treat the case with systemic artery embolization using TAE followed by S6 segmentectomy. Preoperative TAE of the systemic artery was employed to reduce intraoperative bleeding. Microcoils were used as emboli to avoid a risk of using liquid embolization material which can flow into the pulmonary artery through the shunt vessels [14]. However, blood flow from the pulmonary artery was maintained even after embolization of the systemic artery (Fig. 4A, B). In surgery, we preceded the dissection of the pleural adhesion with A6 ligation (Fig. 4C), resulting in minimal intraoperative blood loss. We surmise that S6 segmentectomy minimized the risk of future recanalization of systemic arteries into pulmonary arteries. To our knowledge, the reports describing the treatment of SPAS with TAE and subsequent segmentectomy are very rare.

## Conclusion

We reported a case of SPAS, who was successfully treated with the combination of TAE and subsequent segmentectomy. The blood loss during surgery was minimal and this strategy appeared to minimize future recanalization and hemoptysis. Further studies and long-term follow-up of SPAS patients are required to establish standardized management guidelines for this rare condition.

## Data Availability

All data generated or analysed during this study are included in this published article.

## Abbreviations

CT: Computed tomography
ICG: Indocyanine green
Lt. A6: Left apical segmental pulmonary artery of the left lower lobe
Lt. S6: Left lower lobe superior segment of lung
pAVM: Pulmonary arteriovenous malformations
PE: Pulmonary embolism
SPAS: Systemic-to-pulmonary artery shunt
TAE: Transcatheter arterial embolization
